# HIV-1 Coinfection Profoundly Alters Intrahepatic Chemokine but Not Inflammatory Cytokine Profiles in HCV-Infected Subjects

**DOI:** 10.1371/journal.pone.0086964

**Published:** 2014-02-06

**Authors:** Sishun Hu, Marwan Ghabril, Tohti Amet, Ningjie Hu, Daniel Byrd, Kai Yang, Raj Vuppalanchi, Romil Saxena, Mona Desai, Jie Lan, Raymond Johnson, Samir Gupta, Naga Chalasani, Qigui Yu

**Affiliations:** 1 Department of Microbiology and Immunology, Indiana University School of Medicine, Indianapolis, Indiana, United States of America; 2 Division of Gastroenterology/Hepatology, Department of Medicine, Indiana University School of Medicine, Indianapolis, Indiana, United States of America; 3 Department of Pathology, Indiana University School of Medicine, Indianapolis, Indiana, United States of America; 4 Division of Infectious Diseases, Department of Medicine, Indiana University School of Medicine, Indianapolis, Indiana, United States of America; 5 Center for AIDS Research, Indiana University School of Medicine, Indianapolis, Indiana, United States of America; 6 College of Veterinary Medicine, Huazhong Agricultural University, Wuhan, Hubei, China; 7 Zhejiang Provincial Key Laboratory for Technology & Application of Model Organisms, Wenzhou Medical College, Wenzhou, Zhejiang, China; University of Cincinnati College of Medicine, United States of America

## Abstract

The pathogenesis of accelerated liver damage in subjects coinfected with hepatitis C virus (HCV) and human immunodeficiency virus type 1 (HIV-1) remains largely unknown. Recent studies suggest that ongoing chronic liver inflammation is responsible for the liver injury in HCV-infected patients. We aimed to determine whether HIV-1 coinfection altered intrahepatic inflammatory profiles in HCV infection, thereby hastening liver damage. We used a real-time RT-PCR-based array to comparatively analyze intrahepatic inflammation gene profiles in liver biopsy specimens from HCV-infected (n = 16), HCV/HIV-1-coinfected (n = 8) and uninfected (n = 8) individuals. We then used human hepatocytes to study the molecular mechanisms underlying alternations of the inflammatory profiles. Compared with uninfected individuals, HCV infection and HCV/HIV-1 coinfection markedly altered expression of 59.5% and 50.0% of 84 inflammation-related genes tested, respectively. Among these genes affected, HCV infection up-regulated the expression of 24 genes and down-regulated the expression of 26 genes, whereas HCV/HIV-1 coinfection up-regulated the expression of 21 genes and down-regulated the expression of 21 genes. Compared with HCV infection, HCV/HIV-1 coinfection did not dramatically affect intrahepatic gene expression profiles of cytokines and their receptors, but profoundly altered expression of several chemokine genes including up-regulation of the CXCR3-associated chemokines. Human hepatocytes produced these chemokines in response to virus-related microbial translocation, viral protein stimulation, and antiviral immune responses.

**Conclusions:**

HIV-1 coinfection profoundly alters intrahepatic chemokine but not cytokine profiles in HCV-infected subjects. The altered chemokines may orchestrate the tissue-specific and cell-selective trafficking of immune cells and autoimmunity to accelerate liver disease in HCV/HIV-1 coinfection.

## Introduction

HIV-1 and HCV infections are both major global health problems. The HIV-1 pandemic has claimed over 20 million lives and more than 33 million people are estimated to be living with HIV-1/AIDS worldwide [1]. HCV infects 170 million people globally [2], and the majority of these infected individuals develop chronic hepatitis thereby becoming a potential source of HCV transmission. The number of people living with HIV-1 or HCV continues to grow because no vaccine is currently available to protect against either virus. Due to their shared routes of transmission, HIV-1 and HCV coinfection (HCV/HIV-1) is common, affecting approximately 25–33% of HIV-1-infected persons [3]. HIV-1 coinfection accelerates HCV-related liver injury, resulting in faster development of cirrhosis and end-stage liver disease [4]. The accelerated progression of liver disease due to HCV/HIV-1 coinfection has emerged as a leading cause of death in HIV-1-infected persons. The pathogenic mechanisms as to why HCV-associated liver disease is worse in the presence of HIV-1 remain unknown, but could be related to alternations of liver inflammatory profiles, impaired immune responses, and drug-induced hepatotoxicity.

The liver injury in HCV infection is thought to be mainly due to attacks of immune responses and of inflammatory factors rather than a direct cytopathic effect (CPE) of the virus itself [5]–[7]. Acute HCV infection in humans or chimpanzees shows that high levels of viremia are detectable for several weeks before the onset of hepatitis, suggesting that HCV is not directly cytopathic. Following HCV infection, the immune system responds to viral components by activating immune cells to specifically combat the viral infection. However, when this vigorous immune response fails to eliminate the virus, chronic infection is established. This, in turn, results in an ongoing process of hepatic apoptosis, inflammation, regeneration and fibrosis that in many cases leads to the development of cirrhosis and of hepatocellular carcinoma (HCC). Inflammatory cytokines, chemokines and their receptors play a major role in this active liver damage process [5]–[7]. Among the cytokines, increased intrahepatic expressions of tumor necrosis factor alpha (TNF-α), transforming growth factor beta (TGF-β) and others have been observed in persons with chronic HCV infection [8]. These cytokines have been recognized as key regulators in liver damage [8].

Chemokines that regulate immune cell infiltration and leukocyte trafficking are also important in the development of HCV-related liver injury [9]. In patients chronically infected with HCV, peripheral blood and intrahepatic levels of chemokines including CXCL9, CXCL10, and CXCL11 are elevated compared to uninfected individuals [9]. Importantly, the levels of these chemokines are positively correlated with the degree of hepatic damage [10]–[12]. Interestingly, CXCL9, CXCL10 and CXCL11 are interferon (IFN) inducible and share a unique chemokine receptor CXCR3 found predominantly on activated T cells and natural killer (NK) cells [13], [14]. In chronic HCV infection, elevated levels of intrahepatic CXCL9, CXCL10, and CXCL11 (CXCR3-associated chemokines) foster ongoing intrahepatic inflammation through the attraction of CXCR3^+^ lymphocytes into liver, resulting in development and promotion of liver cell injury and fibrogenesis. Thus, among the large chemokine and chemokine receptor families, CXCR3-associated chemokines are key players in the maintenance and amplification of HCV-related damage processing.

Overall, inflammatory cytokines, chemokines and their receptors play an important role in liver inflammation and disease progression in HCV infection. Whether these inflammatory factors play a critical role in the pathogenesis of accelerated liver damage in subjects coinfected with HCV/HIV-1 has not been clearly defined. The current study was undertaken to compare intrahepatic inflammatory profiles in HCV/HIV-1 coinfection versus HCV infection. We found that HIV-1 coinfection did not dramatically affect intrahepatic gene expression profiles of cytokines and their receptors, but profoundly altered expression of chemokine genes including CXCR3-associated chemokines. The molecular mechanisms underlying the accelerated up-regulation of intrahepatic CXCR3-associated chemokines in HCV/HIV-1 coinfection are associated with HIV-1-related microbial translocation, viral protein stimulation, and antiviral immune responses. Our data provide evidence that chemokines that attract activated immune cells to the liver are important in the progression of accelerated liver disease in HCV/HIV-1 coinfection.

## Materials and Methods

### Study Subjects

Two cohorts (Cohorts 1 and 2) of individuals were included in this study. Cohort 1 patients chronically infected with HCV (16) or HCV/HIV-1 (8) were referred to the clinics of the Indiana University Medical Center between 2003 and 2006 for evaluation of HCV-associated liver diseases (Table 1). Liver biopsy specimens were obtained from these patients. In addition, liver biopsy specimens from 8 HCV-negative/HIV-1-negative (uninfected) liver donors who had brain death due to motor vehicle accidents were also obtained and used as background controls in this study. None of the patients had received anti-HCV therapy within 6 months prior to the liver biopsy. At the time of the liver biopsy, each liver tissue was divided into two pieces, one was placed in 10% neutral buffered formalin and then embedded in paraffin for histological examination, and another was immediately snap frozen in liquid nitrogen for subsequent RNA isolation. Portal, peripheral, and lobular inflammation and fibrosis were evaluated and graded by histological examination in a blinded fashion on all liver biopsy specimens by an experienced liver pathologist according to the Ishak system [15]. Fibrosis was staged into F0 (no fibrosis) to F6 (cirrhosis), whereas necroinflammation was graded into 0 (no inflammation) to 18 (necroinflammation in all portal areas) [15].

**Table 1:** Demographic, clinical, and liver histological features of the cohort 1 patients at the time of liver biopsy.

Patient ID	Age (years)	Gender	Race	HCV type	HCV viral load (IU/mL plasma)	HIV-1 viral load (copies/mL plasma)	CD4 count (numbers/µL blood)	ALP	ALT	AST	Inflammation Grade	Fibrosis score
**HCV**												
C294	51	F	B	1a	>3,500,000	Negative	NA	82	30	30	3	0
C240	54	F	C	1a	875,000	Negative	NA	89	20	21	6	1
C945	42	M	C	1a	783,000	Negative	NA	62	59	42	5	2
C320	52	M	C	1a	>3,500,000	Negative	NA	86	331	150	2	2
C286	51	M	C	1a	842,000	Negative	NA	84	154		5	3
C016	55	M	C	1b	3,115,000	Negative	NA	57	60	68	6	2
C184	51	M	C	1a	3,690,000	Negative	NA	104	111	59	7	1
C631	46	M	C	1b	>3,500,000	Negative	NA	61	151	128	4	2
C921	58	M	C	1b	>3,500,000	Negative	NA	126	44	48	6	1
C200	60	M	B	1a	873,000	Negative	NA	69	35	40	5	3
C050	50	F	C	1a	3,730,000	Negative	NA	268	48	68	8	4
C310	43	M	B	1a	>3,500,000	Negative	NA	101	52	62	8	5
C845	65	M	C	1a	>3,500,000	Negative	NA	124	65	68	10	2
C673	53	M	C	1b	1,440,000	Negative	NA	142	122	154	10	3
C550	57	F	B	1a	1,915,000	Negative	NA	115	68	64	9	4
C147	55	F	C	1a	1,258,000	Negative	NA	112	65	71	8	4
**HCV/HIV-1**												
IC450	42	F	B	1a	3,470,000	11,300	376	66	18	25	5	2
IC167	53	F	B	1a	>3,500,000	<400	1,022	115	52	54	8	5
IC636	50	F	C	1a/b	>10,000,000	7,300	358	51	27	27	5	2
IC344	40	M	C	1a	7,390,000	<400	407	86	70	50	6	1
IC056	41	M	C	1a	>10,000,000	173,000	151	119	187	332	6	0
IC453	45	F	C	1a	3,470,000	11,000	376	70	17	26	3	1
IC756	55	M	B	1a	4,250,000	<50	359	86	29	47	4	2
IC992	44	M	C	1a	>10,000,000	<50	312	140	39	29	3	1
**Normal**												
N022	54	F	C	NA	Negative	Negative	NA	NA	NA	NA	NA	NA
N430	45	F	C	NA	Negative	Negative	NA	NA	NA	NA	NA	NA
N587	46	F	B	NA	Negative	Negative	NA	NA	NA	NA	NA	NA
N700A	34	F	C	NA	Negative	Negative	NA	NA	NA	NA	NA	NA
N865	24	M	B	NA	Negative	Negative	NA	NA	NA	NA	NA	NA
N607	18	F	C	NA	Negative	Negative	NA	NA	NA	NA	NA	NA
N638	55	F	C	NA	Negative	Negative	NA	NA	NA	NA	NA	NA
N835	17	F	C	NA	Negative	Negative	NA	NA	NA	NA	NA	NA

**Note:** ALP, alkaline phosphatase; ALT, alanine aminotransferase; AST, aspartate aminotransferase; NA, not applicable; F, female; M, male; B, black; C, Caucasian.

Peripheral blood was obtained from Cohort 2 individuals infected or uninfected with HCV, HIV-1, or HCV/HIV-1 as described in the Table 2 and our previous reports [16], [17]. These blood samples were separated into peripheral blood mononuclear cells (PBMCs) and plasma that were stored at −80°C until use.

**Table 2:** Demographic and clinical characteristics of the cohort 2 subjects.

	HCV (n = 39)	HIV-1 (n = 37)	HCV/HIV-1 (n = 16)	Uninfected (n = 16)
Mean ages (years)	45 (37–60)	44 (41–46)	42 (33–55)	41 (30–55)
Gender, male (%)	32 (82.1)	29 (78.4)	12 (75)	13 (81.3)
HIV-1-related features		11 (8–14)	10 (7–12)	
Years since HIV-1 diagnosis				
ART naïve (%)		3 (8.1)	1 (6.3)	
CD4 count (numbers/µL)		412 (301–563)	389 (296–542)	
HIV-1 viral load (log_10_copies/mL)		1.5	1.6	
HCV-related features	6 (3–8)		5 (2–7)	
Years since HCV diagnosis				
HCV viral load (log_10_IU/mL)	5.6		5.8	
Peg-IFN/REV therapy (%)	9 (23.1)		4 (25)	
Serum AST (IU/mL)	106 (43–146)		112 (47–155)	
Serum ALT (IU/mL)	87 (53–122)		96 (56–143)	

**Note:** AST, aspartate aminotransferase; ALT, alanine aminotransferase; and Peg-IFN/REV: pegylated IFN-α plus ribavirin.

The study was performed according to a protocol approved by the Indiana University School of Medicine Institutional Review Board. Written informed consent was obtained from each of all participants before specimen collection.

### RT^2^ Profiler PCR Array

Total RNA was extracted from liver biopsy tissues using TRIzol reagent (Invitrogen, Carlsbad, CA), followed by a purification to eliminate genomic DNA, protein and organic contaminations using the RNeasy Mini Kit (Qiagen, Valencia, CA). The concentration and integrity of all RNA samples were assessed using the NanoDrop ND-2000 spectrophotometer (NanoDrop Technologies, Wilmington, DE) and the Bioanalyzer 2100 system (Agilent Technologies, Santa Clara, CA). The same amount of total RNA (800 ng) from each sample was subjected to a 20 µL cDNA synthesis reaction using the RT^2^ First Strand Kit according to the manufacturer’s instructions (SABiosciences, Frederick, MD). The resulting cDNA reaction (20 µL per sample) was diluted in 90 µL of nuclease-free H_2_O (Qiagen, Valencia, CA). The diluted cDNA (102 µL) was mixed with 1,248 µL of H_2_O plus 1,350 µL of 2×SABiosciences SYBR green RT^2^ qPCR Master Mix. The cocktail was dispensed at 25 µL per well into the 96-well RT^2^ Profiler PCR Array plate for profiling a total of 84 inflammation-related genes as described in manufacturer’s instructions (PAHS-011Z, SABiosciences, Frederick, MD). Each array plate also contains five commonly used human housekeeping genes (ACTB, B2M, GAPDH, HPRT1, and RPL13A), and a panel of proprietary control genes to monitor genomic DNA contamination (HGDC) as well as the first strand synthesis (RTC) and real-time PCR efficiency (PPC). The plate was subjected to real-time PCR with a two-step cycling program in an ABI PRISM® 7000 Sequence Detection System: 95°C for 10 min, followed by 40 cycles of 95°C for 15 sec and 60°C for 1 min. The resulting threshold cycle values for all wells were exported to a blank Excel spreadsheet, and then uploaded to the SABiosciences Web for data analysis using the SABiosciences Web-based PCR Array Data Analysis Software version 3.5 (http://www.sabiosciences.com/pcr/arrayanalysis.php). The fold changes of gene expression were calculated in comparison to the values of controls: Fold change = 2(ΔCt [control] −ΔCt [experiment]). Here, ΔCt = Ct (the gene of interest or GOI)−Ct (the housekeeping gene or HKG), where Ct is cycle threshold. To enable comparison between runs, the same threshold was established for all genes and runs. Both RTC and PPC were triplicated and the average of their cycle threshold (Ct) values was used to normalize gene expression and determine fold change between groups. Genes were evaluated on the basis of the criteria of at least a 2-fold up- or down-regulation compared with control and that their regulation was statistically significant at a 95% confidence level (*p* value <0.05) as reported previously [18]. The confidence level was constructed on the data obtained from each sample in triplicate and the sample sizes of healthy donors, HCV-infected or HCV/HIV-1-coinfected individuals.

### Measurement of Plasma Lipopolysaccharide (LPS)

Plasma samples collected from 16 uninfected, 39 HCV-infected, 37 HIV-1-infected, and 16 HCV/HIV-1-coinfected individuals were subjected to LPS measurement using the Limulus Amebocyte Lysate Assay (LAL) (Lonza, Walkersville, MD). Samples were run in duplicates and subjected to readout of optical density at 405 nm (OD405) prior to addition of substrate to obtain background. The background, if present, was subtracted from the final OD405 readout resulting from substrate reaction.

### Stimulation of PBMCs, Monocytes, or T Cells with LPS

PBMCs from healthy blood donors were directly used in this study or subjected to isolation of monocytes and T cells using Dynal Monocyte Negative Isolation Kit (Invitrogen, Grand Island, NY) and Pan T Cell Isolation Kit II (Miltenyi Biotec, Auburn, CA), respectively. The purity of the enriched monocytes or T cells was over 98% as assessed by CD14 plus CD3 staining and flow cytometric analysis (FACS). PBMCs, monocytes or T cells (1×10^6^ cells/mL) were incubated with LPS (Sigma, St. Louis, MO) at various concentrations in complete RPMI 1640 medium at 37°C for 48 h. Cell-free supernatant from LPS-stimulated cells (LPS-S) was subjected to measurement of IFN-γ using the Human IFN-gamma ELISA Kit (RayBiotech, Norcross, GA), or tested for its biological activity in induction of CXCR3-associated chemokines by human hepatocytes.

### CXCR3-associated Chemokine Expression by Huh7.5.1 Cells

The concentrations of CXCR3-associated chemokines in the culture supernatant samples from stimulated or unstimulated Huh7.5.1 cells, an HCV-permissive human hepatic cell line, were measured using the Human MIG/CXCL9, IP-10/CXCL10 and I-TAC/CXCL11 ELISA kits (RayBiotech, Norcross, GA), respectively. Each sample was run in triplicates. Huh7.5.1 cells grown on 24-well plates at 70–80% confluence were treated with LPS (50–1,000 pg/mL), TNF-α (1–40 ng/mL), IFN-γ (0.1–10 ng/mL), IL-1β (0.1–10 ng/mL), 1∶10 diluted LPS-S (diluted in phosphate buffered saline or PBS) or with different combinations at 37°C for 12 h. TNF-α, IFN-γ and IL-1β were purchased from R&D Systems (Minneapolis, MN). Huh7.5.1 cells and supernatant were harvested and subjected to measurement of CXCR3-associated chemokines using FACS and ELISA, respectively.

To block the biological activity of IFN-γ or IL-1β, human IFN-γ-specific neutralizing antibody (nAb) F12, human IL-1β-specific nAb F2 (R&D Systems, Minneapolis, MN), or their combinations were pre-incubated with IFN-γ, IL-1β or LPS-S at 37°C for 30 min and then added to Huh7.5.1 cells. Cells were cultured at 37°C for 12 h. Supernatant was then harvested for measuring CXCR3-associated chemokines as described above.

To study the effects of HIV-1 proteins on production of CXCR3-associated chemokines by hepatocytes, Huh7.5.1 cells were incubated with 1∶10 diluted LPS-S from LPS-stimulated PBMCs plus HIV-1 gp120 or Tat at various concentrations ranging from 0.1 to 10 µg/mL. After 24 h incubation, the cell-free supernatants were harvested and subjected to ELISA to measure CXCR3-associated chemokines. All experiments were performed in triplicates. Both recombinant HIV-1_IIIB_ gp120 and Tat were purchased from Advanced Biotechnologies (Columbia, MD). These proteins were produced using the baculovirus expression system and purified by immunoaffinity chromatography.

### Flow Cytometric Analysis (FACS)

Intracellular staining was performed to enumerate the number of Huh7.5.1 cells producing single, two or all three of CXCL9, CXCL10, CXCL11 in response to IFN-γ stimulation. Intracellular chemokine staining was performed using the Cytofix/Cytoperm kit (BD PharMingen, San Diego, CA). Antibodies against human CXCL9, CXCL10, and CXCL11 (BD Biosciences, San Jose, CA) were used for the staining. Cells were then subjected to FACS analysis using a BD FACSCalibur (BD Biosciences). Data were analyzed using FlowJo software (Tree Star, San Carlos, CA).

### Statistical Analysis

LPS data were reported as the mean ± standard deviations (SD). The differences in plasma LPS levels among the groups were assessed using Holm-Sidak one-way repeated-measures ANOVA (RMANOVA). Statistical significance of CXCR3-associated chemokine expression by hepatocytes in response to different stimulants was analyzed using the Student’s *t-*test. A *p*-value of <0.05 was considered significant.

## Results

### Intrahepatic Inflammatory Profiles are Altered in HCV/HIV-1-Coinfected Individuals Compared to HCV-infected Individuals

We hypothesized that HIV-1 coinfection would exert an inappropriate inflammatory response to worsen HCV liver disease. To test this hypothesis, we used the Human Inflammatory Cytokines & Receptors PCR Array to comparatively analyze intrahepatic inflammatory profiles from 16 HCV-infected, 8 HCV/HIV-1-coinfected, and 8 uninfected subjects. The main demographic, clinical, virological, and histological characteristics of the study subjects at the time of liver biopsy are summarized in Table 1. The liver biochemistries in HCV infected vs. HCV/HIV-1 coinfected subjects were: alkaline phosphatase (ALP) 105±50 IU/mL vs. 92±30 IU/mL (*p = *0.5), alanine aminotransferase (ALT) 88±76 IU/mL vs. 55±53 IU/mL (*p = *0.3), aspartate aminotransferase (AST) 74±41 IU/mL vs. 74±53 IU/mL (*p = *0.9), and total bilirubin 0.9±0.5 vs. 0.8±0.5 mg/dL (*p* = 0.7). The HCV viral load was 2,500,000±1,250,000 IU/mL (ranging from 783,000 to >10,000,000 IU/mL) in HCV infection vs. 6,510,000±3,160,000 (ranging from 3,470,000 to >10,000,000 IU/mL) (*p = *0.0002) in HCV/HIV-1 coinfection (*p* = 0.0002). The histologic Ishak fibrosis stages (F score) in HCV infected vs. HCV/HIV-1 coinfected patients were 2.5±1.4 vs. 1.8±1.5 (*p = *0.3), and the inflammation grades were 6.4±2.4 vs. 5±1.7 (*p = *0.2). Thus, compared to patients infected with HCV only, HCV/HIV-1 coinfected patients enrolled in this study have higher HCV viral loads, but show no significant difference in their liver function, fibrosis and inflammation.

**Figure 1:**
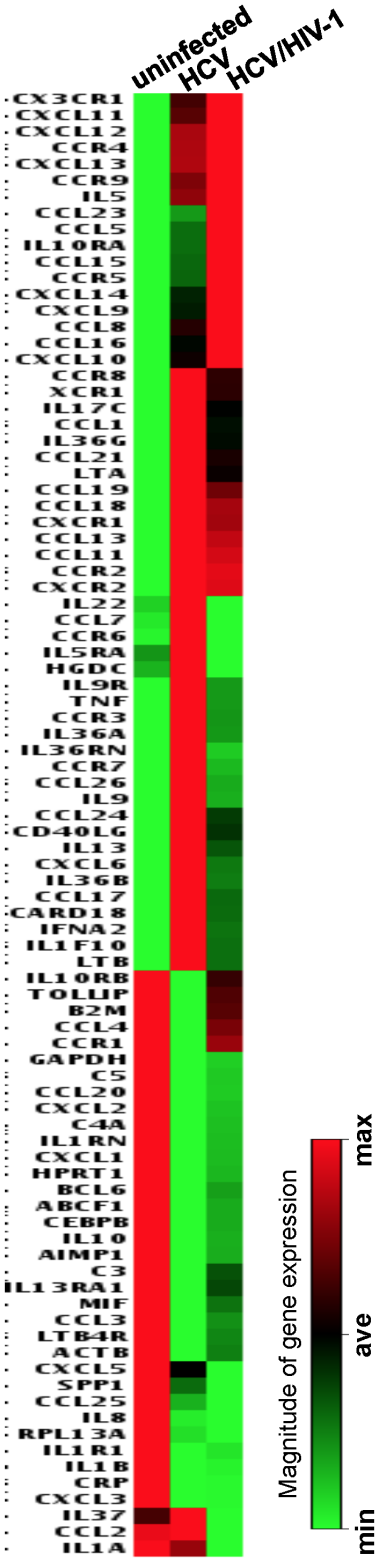
Intrahepatic inflammatory genes with altered expression during HCV/HIV-1 coinfection vs HCV infection. A real-time PCR-based array was used to analyze intrahepatic inflammatory profiles in the liver biopsy specimens from HCV-infected (n = 16), HCV/HIV-1 coinfected (n = 8), and uninfected (n = 8) donors. A heat map illustrates two-dimensional hierarchical clustering of 84 inflammation-related genes including 23 inflammatory cytokine genes, 7 cytokine receptor genes, 31 chemokine genes, 12 chemokine receptor genes, and 11 other genes involved in inflammatory response (PAHS-011Z, SABiosciences, Frederick, MD, ). Genes were identified as differentially regulated in infected vs uninfected individuals by analysis of variance at a 95% confidence level. Increased and decreased expression of specific genes is illustrated by red and green, respectively, while black indicates no change.

A total of 84 inflammation-related genes encoding inflammatory cytokines/receptors, chemokines/receptors and others involved in inflammation were analyzed using the Human Inflammatory Cytokines & Receptors PCR Array. Data were pooled from each group (uninfected, HCV-infected or HCV/HIV-1-coinfected) and were subsequently subjected to the Ward’s hierarchical clustering method to construct a “heat map” at a 99% confidence level (Fig. 1). For each experiment, intrahepatic gene expression from an individual infected with HCV or HCV/HIV-1 was compared to that from the control reference pool of uninfected individuals. Gene expression was evaluated on the basis of the criteria of at least a 2-fold up- or down-regulation compared with controls and that they were regulated in more than 95% of comparisons (*p* value <0.05). Table 3 shows the summary of intrahepatic inflammatory gene expression regulated by HCV infection or HCV/HIV-1 coinfection. Compared with gene expression in liver biopsies from uninfected individuals, HCV infection up-regulated 24 genes (Table 3, Panel A), down-regulated 26 genes (Table 3, Panel B) and had no effect on 34 genes, whereas HCV/HIV-1 coinfection up-regulated 21 genes (Table 3, Panel A), down-regulated 21 genes (Table 3, Panel B) and had no effect on 42 genes. There were 30 genes that were not affected by neither HCV infection nor HCV/HIV-1 coinfection (Table 3, Panel C). Among those genes that were up-regulated, 19 genes were affected by both HCV infection and HCV/HIV-1 coinfection (Panel A, highlighted in bold), 5 additional genes were affected by HCV infection only (Panel A, highlighted in italic), and 2 additional genes were affected by HCV/HIV-1 coinfection only (Panel A, highlighted in italic). Among those genes that were down-regulated, 19 genes were affected by both HCV infection and HCV/HIV-1 coinfection, 7 additional genes were affected by HCV infection only, and 2 additional genes were affected by HCV/HIV-1 coinfection only. Thus, HCV infection and HCV/HIV-1 coinfection alter expression of many of the same genes, while they also differentially regulate a number of different genes.

**Table 3:** Regulation of intrahepatic inflammatory gene expression by HCV infection or HCV/HIV-1 coinfection.

	HCV vs Uninf	HCV/HIV vs Uninf		HCV vs Uninf	HCV/HIV vs Uninf		HCV vs Uninf	HCV/HIV vs Uninf	
Gene	Fold change	Fold change	Gene	Fold change	Fold change	Gene	Fold change	Fold change	
**CCL11**	**3.4**	**3.3**	**BCL6**	**−3.1**	**−2.3**	ABCF1	−1.7	−1.5	
**CCL13**	**2.2**	**2.2**	**C3**	**−4.1**	**−2.0**	CCL1	1.8	1.4	
**CCL17**	**5.3**	**2.3**	**C4A**	**−2.9**	**−2.3**	CCL15	1.1	1.6	
**CCL18**	**2.3**	**2.2**	**C5**	**−3.3**	**−2.6**	CCL16	1.2	1.5	
**CCL19**	**46.80**	**34.90**	**CCL20**	**−5.1**	**−3.6**	CCL2	−1.1	−1.2	
**CCL21**	**7.2**	**4.5**	**CCL25**	**−6.3**	**−38.50**	CCL23	1.1	2.0	
*CCL24*	*2.9*		**CCL3**	**−4.2**	**−2.5**	CCL7	1.4	−1.1	
*CCL26*	*3.3*		*CCL4*	*−2.8*		CCR2	1.2	1.2	
**CCL5**	**2.2**	**5.6**	*CCR1*	*−2.4*		CCR3	1.8	1.1	
**CCL8**	**3.1**	**4.9**	**CEBPB**	**−4.1**	**−2.8**	CCR7	1.5	1.0	
**CCR4**	**2.2**	**2.5**	**CRP**	**−342.700**	**−144.200**	CCR9	1.1	1.2	
*CCR5*		*3.4*	**CXCL1**	**−6.5**	**−3.8**	CARD18	1.8	1.2	
*CCR6*	*2.0*		**CXCL2**	**−17.20**	**−6.3**	IFNA2	1.8	1.2	
*CCR8*	*2.2*		**CXCL3**	**−8.0**	**−7.4**	IL10RA	1.0	1.3	
**CX3CR1**	**2.6**	**3.6**	*CXCL5*		**−** *3.0*	IL13	2.0	1.3	
**CXCL9**	**8.3**	**17.90**	**IL10**	**−3.9**	**−2.7**	IL17C	1.5	1.2	
**CXCL10**	**58.30**	**112.600**	*IL10RB*	**−** *2.6*		IL1F10	1.8	1.2	
**CXCL11**	**12.90**	**19.30**	*IL13RA1*	**−** *2.9*		IL36RN	1.6	1.0	
**CXCL12**	**2.4**	**2.8**	*IL1A*		**−** *3.5*	IL36A	1.8	1.1	
**CXCL13**	**2.3**	**2.7**	**IL1B**	**−4.7**	**−4.3**	IL37	1.1	**−**1.6	
**CXCL14**	**2.8**	**5.5**	**IL1R1**	**−2.4**	**−2.2**	IL36B	1.9	1.2	
*CXCL6*	*2.1*		**IL1RN**	**−8.1**	**−4.4**	IL36G	1.1	1.0	
*IL5*		*2.1*	**IL8**	**−7.4**	**−8.8**	IL22	1.9	**−**1.2	
**LTA**	**5.2**	**3.2**	*LTB4R*	**−** *2.2*		IL5RA	1.6	**−**1.3	
**LTB**	**5.8**	**2.4**	**MIF**	**−4.7**	**−2.4**	CXCR1	1.8	1.7	
**CD40LG**	**4.4**	**2.4**	*AIMP1*	**−** *2.3*		CXCR2	1.9	1.9	
Gene No. affected	24	21	**SPP1**	**−2.8**	**−8.2**	IL9	1.8	1.1	
			*TOLLIP*	**−** *2.3*		IL9R	1.8	1.1	
			Gene No. affected	26	21	TNF	1.5	1.1	
						XCR1	1.9	1.6	
						Gene No. affected	30	30	

Note.

**Uninf:** uninfected.

**HCV/HIV:** HCV/HIV-1 coinfection.

**Genes highlighted in bold:** Expression alternations of these genes were observed in both HCV infection and HCV/HIV-1 coinfection.

**Gene No. affected:** total numbers of genes affected or unaffected by either HCV infection or HCV/HIV-1 coinfection.

Notably, genes expressing CXCR3-associated chemokines (CXCL9, CXCL10 and CXCL11), CCL19, and CCL21 were the most frequently up-regulated genes in both HCV infection and HCV/HIV-1 coinfection (Table 3, Panel A), whereas CRP, CCL25 and CXCL2 were the most frequently down-regulated genes in both HCV infection and HCV/HIV-1 coinfection (Table 3, Panel B). Among these genes, HCV/HIV-1 coinfection further up-regulated expression of genes of CXCR3-associated chemokines (Table 3, Panel A), and down-regulated expression of CCL25 gene when compared with HCV infection (Table 3, Panel B).

### HCV/HIV-1 Coinfection Further Enhances Intrahepatic CXCR3-Associated Chemokines

**Figure 2:**
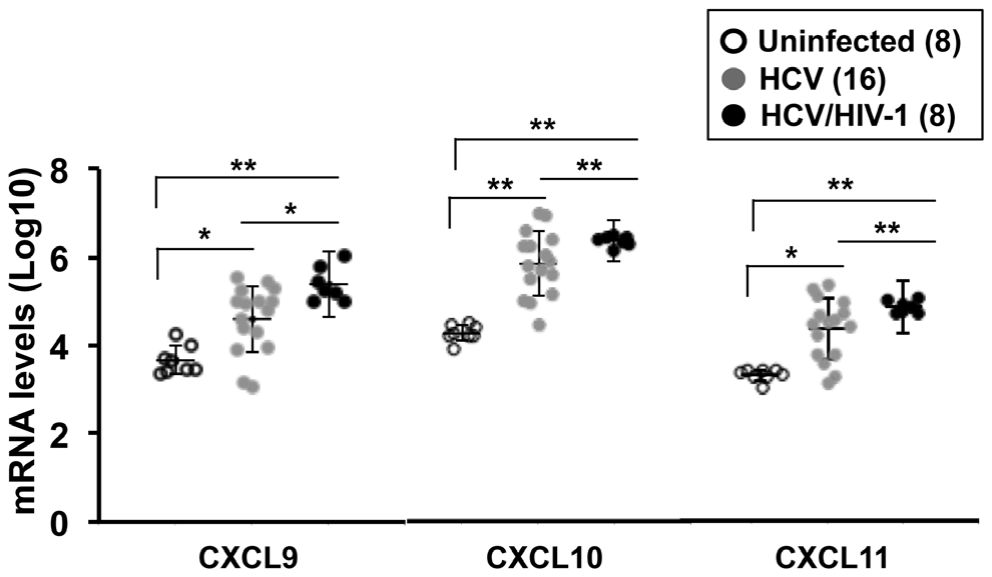
Intrahepatic mRNA levels of CXCR-3-associated chemokines in individuals infected or uninfected with HCV or HCV/HIV-1. Chemokine mRNA levels were measured by qPCR assays. Plasmid constructs containing either partial CXCL9, CXCL10, or CXCL11 gene were used to establish standard curves in each qPCR performance for quantitative analysis of CXCL9, CXCL10, or CXCL11 cDNA copy number. Plasmid DNA concentrations were determined using a ND-1000 UV–vis Spectrophotometer (NanoDrop Technologies, Wilmington, DE). The number of construct copies in the plasmid solution was calculated, based on plasmid vector size (pCR2.1-TOPO, 3931 bp, Invitrogen, Carlsbad, CA) and insert size of CXCL9, CXCL10, or CXCL11 gene fragment, respectively. A plasmid-based calibration curve was generated with 10-fold serial dilutions of plasmid containing the CXCL9, CXCL10, or CXCL11 gene fragment sequence; to control pipetting steps, three 10-fold serial dilutions were prepared, and concentrations were checked by real-time PCR. The Y-axis indicates the logarithmic-transformed levels for each chemokine. The lines in each plot show the mean ± standard deviations (SD). The number of subjects per group is indicated in parenthesis. *, *p*<0.05; **, *p*<0.01.

The real-time RT-PCR-based array revealed that intrahepatic mRNA expression of all three CXCR3-associated chemokines (CXCL9, CXCL10, and CXCL11) was markedly increased in HCV-infected patients when compared with that of liver biopsies from uninfected donors, and the expression of these genes was further up-regulated in HCV/HIV-1 coinfection when compared with that in HCV infection. These alterations were validated and confirmed by quantitative real-time RT-PCR (qPCR). Median intrahepatic CXCL10, CXCL11, and CXCL9 mRNA relative expression levels as expressed in Log10 were 3.66±0.32 (n = 8), 4.29±0.18 (n = 8), and 3.33±0.13 (n = 8) in uninfected controls, and 4.6±0.76 (n = 16), 5.88±0.73 (n = 16), and 4.37±0.69 (n = 16) in chronic HCV patients, respectively (Fig. 2). Compared to HCV infection, HCV/HIV-1 coinfection further increased intrahepatic mRNA expression of all three CXCR3-associated chemokines. Median intrahepatic CXCL10, CXCL11, and CXCL9 mRNA relative expression levels as expressed in Log10 were 5.37±0.39 (n = 8), 6.41±0.11 (n = 8), and 4.88±0.14 (n = 8) in HCV/HIV-1 coinfected patients, respectively (Fig. 2). Notably, CXCL10 was the most markedly elevated in HCV-infected or HCV/HIV-1-coinfected patients when compared with either CXCL9 or CXCL11. These data are consistent with that obtained by the real-time PCR array. Studies have shown that peripheral levels of CXCR3-associated chemokines, particularly CXCL10, are significantly associated with liver fibrosis in chronic HCV-infected patients [9]–[12]. Our data are consistent with these reports. However, more samples from patients at different stages of liver fibrosis are required to determine whether the levels of CXCR3-associated chemokines, particularly CXCL10, are able to serve as biomarkers of liver disease severity in HCV/HIV-1 infection.

### Cytokines Induce Production of CXCR3-Associated Chemokines by Human Hepatocytes

**Figure 3:**
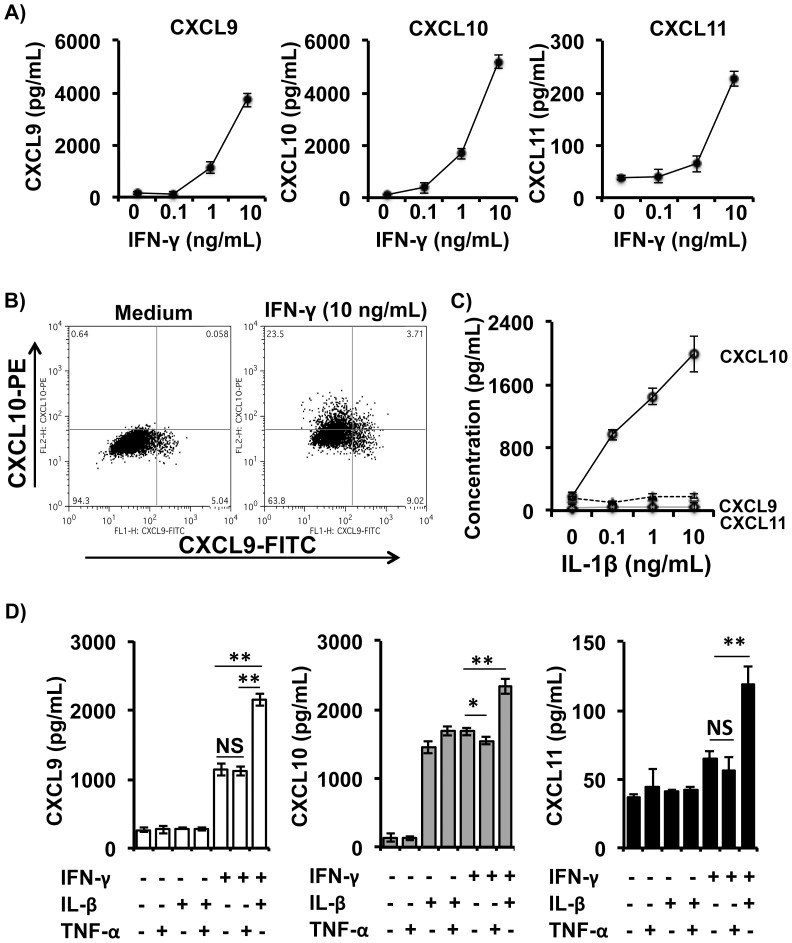
Human hepatocytes produce CXCR3-associated chemokines in response to various stimulants. Huh7.5.1 cells in 24-well plates were treated or untreated with IFN-γ, IL-1β, TNF-α or their combinations at various concentrations for 24 h at 37°C. Cell-free supernatant and cells were subjected to measurement of expression of CXCL9, CXCL10, and CLCL11 using ELISA and FACS, respectively. A) IFN-γ induced Huh7.5.1 cells to produce CXCL9, CXCL10, and CXCL11 in a dose-dependent manner. B) FACS analysis of intracellular CXCL9, CXCL10, and CXCL11 levels in Huh7.5.1 cells in response to IFN-γ stimulation at 10 ng/mL. C) CXCL10, but not CXCL9 or CXCL11, was produced by Huh7.5.1 cells in response to IL-1β treatment in a dose-dependent manner. D) IL-1β, but not TNF-α, enhanced IFN-γ activity in induction of CXCR-associated chemokines by Huh7.5.1 cells. Error bars represent mean ± SD of values from triple wells in each dose. Note that different Y-axis values among CXCL9, CXCL10 and CXCL11 are used. NS, not significant; *p*≥0.05; **, *p*<0.01; and *, *p*<0.05.

All three CXCR3-associated chemokines (CXCL9, CXCL10, and CXCL11) are IFN-γ inducible, and bind to their shared receptor CXCR3 to mediate the chemotaxis of activated immune cells. We found that Huh7.5.1 cells, a human hepatocyte cell line that supports replication of HCV *in vitro*, produced high levels of all three CXCR3-associated chemokines in response to IFN-γ stimulation in a dose-dependent manner (Fig. 3A). Among the cells that responded to IFN-γ stimulation, the majority of cells produced CXCL10 only (Fig. 3B), while a smaller percentage of cells produced CXCL9 (Fig. 3B) or CXCL11 (data not shown). Interestingly, Huh7.5.1 cells were capable of simultaneously producing two or all of these three CXCR3-associated chemokines in response to IFN-γ stimulation (Fig. 3B). Upon stimulation with IFN-γ at 10 ng/mL for 24 h, approximately 3.7% of Huh7.5.1 cells simultaneously produced both CXCL9 and CXCL10 (Fig. 3B), while 23.5% and 9% of the cells produced CXCL10 and CXCL9, respectively (Fig. 3B). These results indicate that Huh7.5.1 cells are heterogeneous and able to differentially respond to IFN-γ stimulation in the context of CXCR3-associated chemokine production.

In contrast to IFN-γ, IL-1β-stimulated Huh7.5.1 cells produced CXCL10 in a dose-dependent manner, but did not affect production of neither CXCL9 nor CXCL11 (Fig. 3C), whereas TNF-α had no effect on the production of any of these chemokines (Fig. 3D). In addition, IL-1β markedly enhanced IFN-γ-induced production of all three CXCR3-associated chemokines, whereas TNF-α did not show any effect on IFN-γ activity in induction of any of them (Fig. 3D). IL-1β, but not TNF-α, had a synergistic effect with IFN-γ on induction of CXCL9, CXCL10, and CXCL11 (Fig. 3D).

### LPS Is Increased in HCV/HIV-1 Coinfected Individuals and It Indirectly Stimulated Hepatocytes to Produce CXCR3-Associated Chemokines

**Figure 4:**
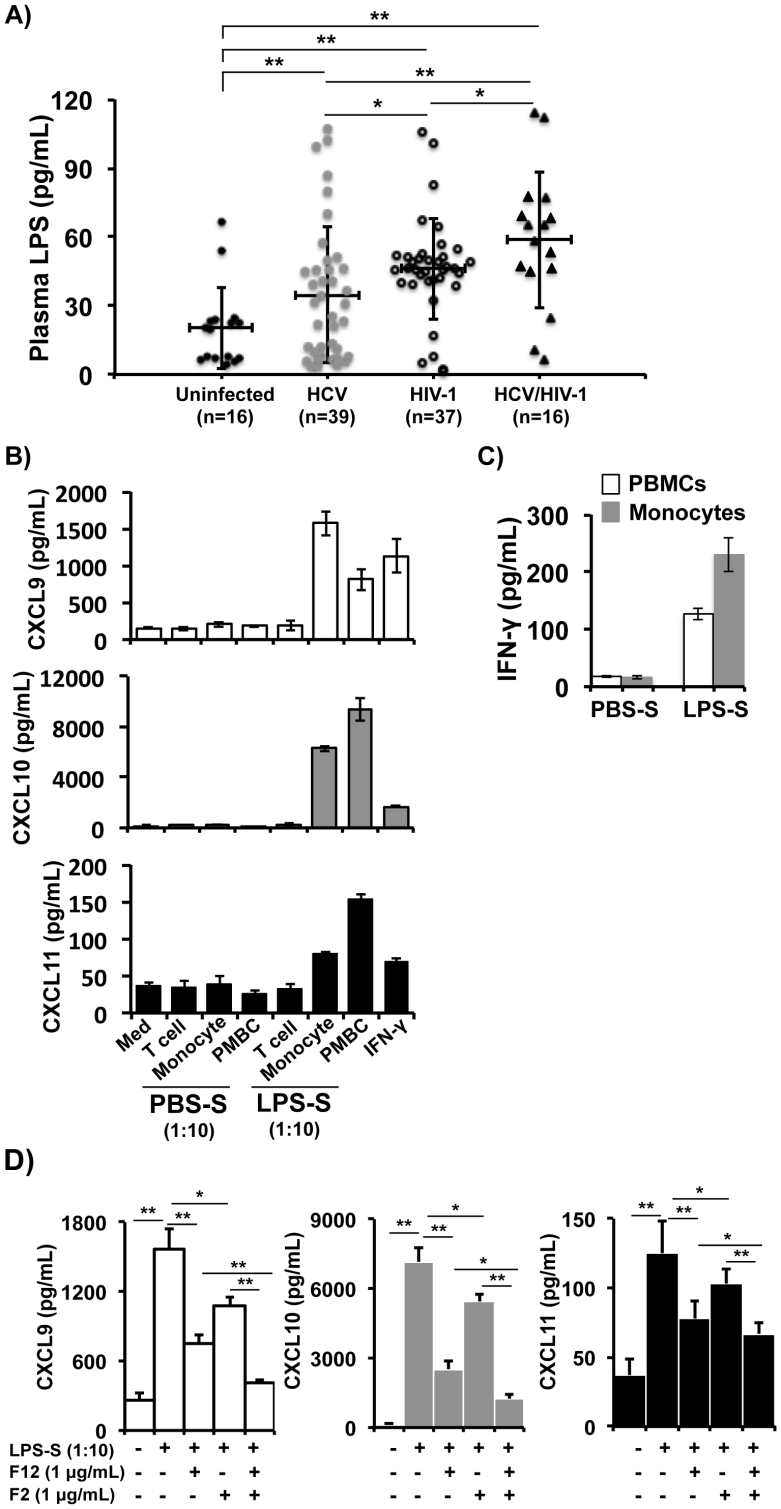
Indirect effect of LPS on production of CXCR3-associated chemokines by human hepatocytes. A) LPS levels in plasma from subjected uninfected, or infected with HCV, HIV-1, or HCV/HIV-1 were determined using the QCL-1000 Chromogenic LAL assay. Lines in each plot represent mean ± SD of values from each group. B) Huh7.5.1 cells produced CXCL9, CXCL10 and CXCL11 in response to stimulation with 1∶10 diluted cell-free supernatant from LPS- or mock-treated pan T cells, monocytes, or PBMCs. IFN-γ at 1 ng/mL was used as a positive control of stimulation. Cell-free supernatant was subjected to ELISA assay to determine levels of CXCL9, CXCL10 and CXCL11. C) Levels of IFN-γ in the supernatant of monocytes or PBMCs treated with LPS or PBS were determined by the ELISA assay. D) LPS-S-mediated enhancement of CXCL9, CXCL10 and CXCL11 production by Huh7.5.1 cells was dependent on IFN-γ and IL-1β. LPS-S-mediated enhancement of CXCL9, CXCL10 and CXCL11 production by Huh7.5.1 cells was significantly blocked by IFN-γ nAb F12, and further down-regulated by IFN-γ nAb F12 (1 µg/mL) plus IL-1β nAb F2 (1 µg/mL). Horizontal bars represent mean ± SD of chemokine protein levels from triple wells of each dose. Note that different Y-axis values among CXCL9, CXCL10 and CXCL11 are used. LPS-S, supernatant from LPS-stimulated PBMCs. *p*<0.05 indicates significant differences. **, *p*<0.01; and *, *p*<0.05.

HIV-1 infection causes severe CD4^+^ T cell depletion in gut-associated lymphoid tissues (GALTs), resulting in severe defects in intestinal epithelial barrier function and integrity [19]. A leaky gut allows poorly invasive enterobacteria to translocate from the intestinal lumen into the systemic circulation [19]. Increased circulating levels of LPS have been demonstrated in HIV-1-infected patients, and display a positive correlation with activation of a systemic immune response, in turn playing a key role in the pathogenesis of HIV-1 disease progression [20]. Similarly, HCV infection also causes a high degree of microbial translocation and elevated plasma LPS levels are strongly correlated with the severity of liver disease [21]. We then asked whether plasma levels of LPS in HCV/HIV-1 coinfected patients were further increased, subsequently stimulating hepatocytes to accelerate production of intrahepatic CXCR3-associated chemokines in these patients. We measured LPS concentrations in plasma samples obtained from 39 HCV-infected, 37 HIV-1-infected, 16 HCV/HIV-1-coinfected, and 16 uninfected blood donors (Table 2). At the time of blood draw, the majority of patients infected with HCV or HCV/HIV-1 were anti-HCV therapy naïve, whereas the vast majority of patients infected with HIV-1 or HCV/HIV-1 were treated with antiretroviral therapy (ART) for years. As shown in Fig. 4A, the mean levels of plasma LPS from HCV-, HIV-1-, and HCV/HIV-1-infected patients were 34.6±29.7 pg/mL, 48.4±8.6 pg/mL, 63.7±49.3 pg/mL, respectively, which were significantly higher than that of plasma from uninfected donors (20.2±17.6 pg/mL). Comparative analysis showed that plasma LPS levels were significantly increased in HCV/HIV-1 coinfection than that of either HCV or HIV-1 infection (Fig. 4A).

We then investigated whether LPS directly or indirectly affected the function of human hepatocytes in the context of CXCR3-associated chemokine production. Human hepatocytes express both Toll-like receptor 4 (TLR4) and CD14, two receptors of LPS, on the cell surface and are directly responsive to LPS stimulation (0.01 to 10 µg/mL) to produce IL-6 [22], [23]. We used LPS at concentrations of 0.01 to 10 µg/mL to directly treat Huh7.5.1 cells for 6–24 h, and did not find induction of any of the three CXCR3-associated chemokines (data not shown), indicating that LPS is unable to directly stimulate hepatocytes to produce these chemokines. We then tested whether LPS indirectly affected hepatocytes through activation of immune cells. LPS at 0.01 µg/mL was used to stimulate PBMCs, monocytes or T cells isolated from uninfected blood donors. The cell-free supernatant (LPS-S) harvested at 24–48 h post stimulation from these conditions was used to culture Huh7.5.1 cells to induce CXCR3-associated chemokine production. As shown in Fig. 4B, LPS-S from stimulated monocytes and PBMCs, but not from T cells, activated Huh7.5.1 cells to produce all three CXCR3-associated chemokines, whereas supernatant from control groups treated with PBS did not stimulate Huh7.5.1 cells to produce any of these chemokines. LPS-S activity in induction of CXCL10 and CXCL11 was not correlated with the concentration of IFN-γ in these supernatant samples. Supernatant from LPS-treated monocytes contained 230±30 pg/mL of IFN-γ that was higher than that in LPS-S from PBMCs (127±10 pg/mL) (Fig. 4C). However, LPS-S from PBMCs stimulated Huh7.5.1 cells to produce higher levels of CXCL10 and CXCL11 than that by LPS-S from monocytes (Fig. 4B). In addition, LPS-S activity in induction of CXCR3-associated chemokines was only partially diminished by IFN-γ nAb F12 at the concentration that can completely block IFN-γ effect (Fig. 4D). These results indicates that factors other than IFN-γ also exist in the LPS-S from PBMCs and may directly stimulate or synergize with IFN-γ to stimulate Huh7.5.1 cells to produce CXCR3-associated chemokines.

We then tested whether LPS-S stimulated Huh7.5.1 cells through IL-1β as this cytokine was also found to be an important mediator in the induction of CXCR3-associated chemokines by hepatocytes (Fig. 3D). We found that LPS-S-mediated induction of CXCR3-associated chemokines was mostly blocked by IFN-γ nAb F12 plus IL-1β nAb F2 when compared with that of each individual Ab treatment (Fig. 4D). Thus, IL-1β in the LPS-S is able to directly stimulate or synergize with IFN-γ to stimulate Huh7.5.1 cells to produce CXCR3-associated chemokines.

### HIV-1 Proteins Enhanced the Activity of LPS-S in Inducing the CXCR3-associated Chemokine Expression by Human Hepatocytes

**Figure 5:**
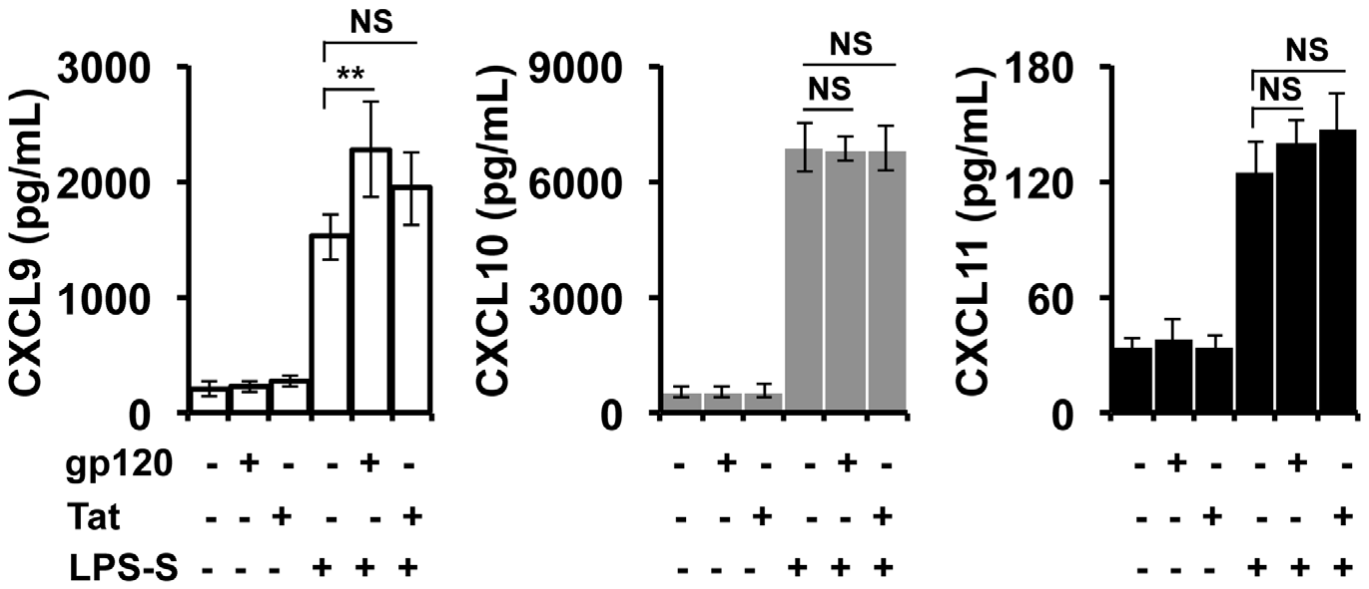
HIV-1 proteins enhance LPS-S activity in induction of CXCR3-associated chemokine by human hepatocytes. Huh7.5.1 cells in 24-well plates were treated with HIV-1 gp120 (1.0 µg/mL) or Tat (1.0 µg/mL) in the presence or absence of LPS-S from LPS-stimulated PBMCs (1∶10 dilution) for 24 h. After stimulation, cell-free supernatant was harvested and then subjected to an ELISA assay to determine protein levels of CXCL9, CXCL10, and CXCL11. Horizontal bars represent mean ± SD of chemokine protein levels from triple wells of each dose. Note that different Y-axis values among CXCL9, CXCL10 and CXCL11 are used. *p*<0.05 indicates significant differences. NS, not significant, **, *p*<0.01; and *, *p*<0.05.

*In vitro* and *in vivo* studies have shown that soluble HIV-1 proteins gp120 and Tat are able to directly stimulate astrocytes to produce CXCL10 [22]. However, we found that neither gp120 nor Tat protein at various concentrations (0.1–10 µg/mL) was able to directly stimulate human hepatocytes to produce any of these three CXCR3-associated chemokines. As shown in Fig. 5, none of the three chemokines was increased in the supernatant of Huh7.5.1 cells post-incubation with gp120 or Tat at 1 µg/mL for up to 24 h when compared with that in controls. These data were consistent with a previous report that CXCL10 was not increased in the supernatant of Huh7.5.1 cells stimulated with HIV-1 Tat at 1 µg/mL for 24 h [24].

We next sought to determine whether HIV-1 proteins were able to cooperate with LPS-S in promoting production of CXCR3-associated chemokines by human hepatocytes. Huh7.5.1 cells were treated with 1∶10 diluted LPS-S plus gp120 or Tat at various concentrations (0.1–10 µg/mL) for 24 h and cell-free supernatant was subjected to measurement of CXCR3-associated chemokines. As shown in Fig. 5, gp120 at concentrations of 1 µg/mL or higher significantly enhanced the LPS-S-mediated induction of CXCL9, as CXCL9 concentration in the combined doses of LPS-S plus gp120 (1 µg/mL) were 2276±413 pg/mL (n = 6) that was significantly greater than the sum of their individual effects (LPS-S: 1519±198 pg/mL, gp120, 225±49, n = 6) (*p*<0.05) (Fig. 5). However, HIV-1 gp120 did not significantly affect LPS-S activity in the induction of CXCL10 and CXCL11. Tat did not co-operate with LPS-S in the induction of any of these three chemokines (Fig. 5). Thus, HIV-1 envelope protein (gp120), and probably HCV envelope proteins as well, accelerated production of CXCL9 in liver tissues of HCV/HIV-1 coinfected patients. This synergistic effect of HIV-1 gp120 with LPS-S on induction of CXCL9 is unlikely resulted from direct action on IFN-γ-dependent pathway because IFN-γ-inducible CXCL10 and CXCL11 were not affected. Our results are in agreement with a previous report that the gene expression of the CXCR3-associated chemokines by astrocytes can be differentially regulated *in vivo* and *in vitro* [24].

## Discussion

In this study, we used a real-time PCR-based array to comparatively analyze intrahepatic inflammatory profiles of HCV/HIV-1-infected versus HCV-infected versus uninfected subjects. The results indicate that the expression profiles of intrahepatic inflammation-related genes are markedly altered in HCV infection and HCV/HIV-1 coinfection when compared with uninfected subjects. Compared with HCV infection, HCV/HIV-1 coinfection further altered the expression of several chemokine genes including up-regulation of the CXCR3-associated chemokines, and down-regulation of CCL25, but did not strikingly accelerate expression of inflammatory cytokines. In fact, intrahepatic mRNA levels of some inflammatory cytokines such as TNF-α, IFN-α, and IL-17 were slightly lower for HCV/HIV-1 coinfected compared to HCV-infected patients (Table 3). These data are consistent with previous reports that intrahepatic cytokine expression is down-regulated during HCV/HIV-1 coinfection [25]–[27], and also with the inflammation grades of liver tissue histology that showed no difference of liver inflammation between HCV/HIV-1 infection and HCV infection (Table 1). Collectively, our data demonstrate that HCV/HIV-1 coinfection does not dramatically affect intrahepatic gene expression profiles of cytokines and their receptors, but profoundly alters the expression of chemokines.

Our data demonstrate that intrahepatic CXCR3-associated chemokines are the most markedly elevated in HCV infection, and are further elevated in HCV/HIV-1 coinfection. These chemokines may play a central role in the pathogenesis of the accelerated liver injury in HCV/HIV-1 coinfection as the levels of these chemokines are correlated with liver fibrosis in HCV infection [28]. A recent report has demonstrated that HIV-1-specific T cells are accumulated in the liver during HCV/HIV-1-coinfection [29], suggesting that intrahepatic chemokines play a significant role in the chemotaxis of these T cells to the liver. These activated HIV-1-specific T cells represent a major effector arm of the cellular immune responses against viral infection, but may also exert a cytotoxic bystander effect on liver cells. We thus focused our efforts on understanding the molecular mechanism underlying the accelerated liver disease in HCV/HIV-1-coinfected patients by studying the regulation of the CXCR3-associated chemokines. We hypothesized that the elevated levels of intrahepatic CXCR3-associated chemokines were associated with HIV-1-related microbial translocation, viral protein stimulation, and antiviral immune responses. In agreement with previous studies, we found that patients infected with either HCV or HIV-1 had elevated levels of plasma LPS when compared with that in uninfected individuals, indicating that both HCV and HIV-1 infections cause a high degree of microbial translocation. We also found that plasma LPS levels were further elevated in HCV/HIV-1 coinfection than that of either HCV or HIV-1 alone (Fig. 4A), indicating that HCV/HIV-1 coinfection causes more rampant microbial translocation. However, LPS did not directly stimulate hepatocytes to produce CXCR3-associated chemokines, although LPS receptors including TLR4 and CD14 are constitutively expressed on the surface of these cells [23]. In contrast, LPS indirectly stimulated hepatocytes to produce CXCR3-associated chemokines through activation of immune cells including monocytes and non-T cells. These cells produced IFN-γ and IL-1β that, in turn, triggered hepatocytes to produce CXCR3-associated chemokines. Significantly, IFN-γ and IL-1β showed a synergistic effect on the induction of CXCR3-associated chemokines by hepatocytes. Therefore, we conclude that both bacterial translocation (LPS) and immune response (IFN-γ and other mediators) can directly or indirectly drive hepatocytes to produce chemokines that attract more activated immune cells into the liver to accelerate liver damage in HCV/HIV-1 coinfection.

In patients coinfected with HCV/HIV-1, HCV and HIV-1 viral loads can be as high as >10,000,000 IU/mL and >173,000 RNA copies/mL of plasma, respectively (Table 1). These virus particles and viral components, particularly HIV-1 proteins, may also directly or indirectly stimulate hepatocytes to produce CXCR3-associated chemokines, as *in vitro* and *in vivo* studies have shown that soluble HIV-1 proteins gp120 and Tat are able to directly stimulate astrocytes to produce CXCL10 [22]. However, we found that neither gp120 nor Tat protein was able to directly stimulate human hepatocytes to produce any of these three CXCR3-associated chemokines (Fig. 5). These data are consistent with a previous report that CXCL10 is not increased in the supernatant of Huh7.5.1 cells stimulated with HIV-1 Tat [24]. However, gp120, but not Tat, enhanced the biological activity of soluble mediators released from LPS-primed immune cells in the induction of CXCL9 by hepatocytes (Fig. 5). Thus, HIV-1 proteins, and probably HCV proteins as well, accelerate intrahepatic chemokines in HCV/HIV-1 coinfected patients.

In conclusion, HIV-1 coinfection alters intrahepatic chemokine profiles in HCV-infected subjects due to multiple factors including virus-related microbial translocation, viral protein stimulation, and antiviral immune responses. All these factors directly or indirectly stimulate hepatocytes to produce high levels of intrahepatic chemokines that attract activated immune cells or enhance autoimmune responses to worsen liver injury. Thus, the chemokines and their receptors orchestrate the tissue-specific and cell type-selective trafficking of immune cells to accelerate liver disease in HCV/HIV-1 coinfection. These molecules should be primary targets for therapeutic interventions in viral liver diseases, particularly in liver injury in HCV/HIV-1 coinfection. Further studies, with larger sample sizes, are warranted to extend these findings. In addition, the other major cell types in the liver including hepatic stellate cells and Kupffer cells should also be studied as these cells are affected by bacterial components as well. Furthermore, immunohistochemistry data are desirable to particularly clarify: (1) correlation of intrahepatic levels of chemokines with liver inflammation and fibrosis; (2) liver cell types producing these chemokines; and (3) colocalization of chemokine-producing liver cells with virus-specific T cells, NK cells and other activated immune cells, as described in HCV infection alone [10]–[12], [30].
